# Alcohol Consumption Among Racial/Ethnic Minorities: Theory and Research

**Published:** 1998

**Authors:** Raul Caetano, Catherine L. Clark, Tammy Tam

**Affiliations:** Raul Caetano, M.D., Ph.D., is a professor and assistant dean of the Dallas Master in Public Health Program, School of Public Health, University of Texas, Dallas, Texas. Catherine L. Clark, Ph.D., is an associate scientist and Tammy Tam, Ph.D., is a scientist at the Alcohol Research Group, Public Health Institute, Berkeley, California

**Keywords:** AOD use pattern, AOD consumption, ethnic differences, minority group, racial differences, Hispanic, African American, Asian American, Native American, hereditary factors, sociocultural norms, causes of AODU (alcohol and other drug use), psychological stress, history of AOD use, literature review

## Abstract

Ethnic minorities (e.g., Hispanics, blacks, Asian-Americans, and Native Americans) are still underrepresented in alcohol research in the United States. Furthermore, existing studies often do not take into consideration the variability that exists within each ethnic group, resulting in inaccurate generalizations. Studies among Hispanics have found substantial differences among Hispanic subgroups in drinking patterns and rates of alcohol-related problems. Moreover, no single variable can explain the observed patterns. Similarly, numerous factors have been shown to shape drinking patterns among blacks, including individual and environmental characteristics as well as historical and cultural factors. Different subgroups of Asian-Americans also vary substantially in their rates of drinking and heavy drinking, although their lifetime alcohol use is lower than the national average. Genetic and cultural factors, as well as stress and historic experiences, may influence drinking patterns of Asian-Americans. The widely differing drinking patterns among Native Americans also are likely shaped by a variety of influences.

Much of the research on alcohol-related issues in the United States has been conducted with samples of whites and has ignored the potential influence of cultural factors, such as race and ethnicity. For example, although nationwide household alcohol surveys in the United States have been administered since 1964, the first national alcohol survey with an emphasis on blacks and Hispanics was implemented only in 1984. Over the past decade, however, considerable advances—primarily in the quality of the research—have been made in alcohol studies among ethnic minorities in the United States. For example, these studies now emphasize more strongly the conceptualization of research questions and employ more sophisticated methodological tools.

The importance of conducting alcohol research among minorities is underscored by findings that members of many ethnic minorities in the United States report higher rates of heavy drinking and alcohol-related problems than do whites (for a review, see Caetano and Clark 1998*a*,*b*). Consequently, it is imperative, from a public health perspective, to better understand ethnic-specific drinking patterns and their associated problems.

A dominant theme that has emerged in analyses of drinking patterns among members of various ethnic minorities is the influence of stressors related to social adjustment to the dominant U.S. culture. Those stressors include the following (Al-Issa 1997):

- Acculturative^1^ stress, which is most typically felt by immigrants who are faced with the turmoil of leaving their homeland and adapting to a new society
- Socioeconomic stress, which is often experienced by ethnic minorities who feel disempowered because of inadequate financial resources and limited social class standing
- Minority stress, which refers to the tensions that minorities encounter resulting from racism.

Although some overlap exists among these stressors, they are conceptually and empirically distinct forces and often require specific coping strategies.

The concept of linking social-adjustment stressors to drinking patterns is partially based on two theories: Durkheim’s (1933) theory of anomie and Leighton’s (1968) theory of mental illness and social disintegration. According to Durkheim’s theory, rapid cultural change causes a condition called anomie—the absence within a society of common social norms and controls. Under those conditions, people lack clear behavioral guidelines, possibly resulting in self-destructive tendencies (e.g., depression and alcohol abuse, which may result in alcohol dependence). Similarly, Leighton (1968) proposed, based on fieldwork observation, that social disintegration and lack of social cohesion precipitate psychological distress and mental illness. He argued that rapid social change and disruptions (e.g., low and unstable income, conflicting cultural values, and fragmented communication networks) cause high stress levels that can result in deviant behaviors and psychological disorders.

A second fundamental theme emerging from recent ethnicity-focused alcohol research is that tremendous variability (i.e., heterogeneity) exists within each ethnic group, posing significant theoretical and methodological challenges to researchers. (For more information on the methodological approaches and challenges associated with research among minorities, see sidebar, pp. 239–241.) As a result of this heterogeneity, broad categorizations of ethnicity, such as “Hispanic” and “Native American,” may lead to inaccurate generalizations and invalid findings. In fact, Phinney (1996) has argued that ethnicity by itself cannot explain such behaviors as drinking patterns and should be used only to describe the common experiences and values held by broad groupings of people of the same race and culture of origin.

**Methodological Approaches for Implementing Research Among Ethnic Minorities**

Studies investigating the drinking practices among ethnic minorities face special challenges at many stages of the research process. These challenges make the research more complex, more expensive, and more difficult to undertake than studies among a majority group. Some of the unique issues that researchers must consider include ethnic group identification, subject selection, instrument development, and data collection and subject recruitment.

***Ethnic Group Identification***

The members of all major ethnic minorities in the United States are highly diverse with respect to their cultural backgrounds:

- Most Hispanics living in the United States have origins in Mexico, Puerto Rico, Cuba, and Central and South America. All of these regions have distinct cultural backgrounds.
- Blacks living in the United States include U.S.-born African-Americans as well as immigrants from such diverse areas as the Caribbean, Africa, and Europe.
- Asian-Americans also originate from many areas, including China, Japan, the Philippines, Korea, Vietnam, Cambodia, Laos, and Hawaii and other Pacific Islands.
- Native Americans belong to hundreds of different tribes with a plethora of distinct languages.

In light of this enormous heterogeneity within ethnic groups, the concept of ethnicity has been the subject of considerable discussion in the alcohol literature. Operational definitions of ethnicity have varied considerably, resulting in imprecise and unreliable comparisons across studies (Heath 1991). Moreover, the uniformity created by grouping people with diverse cultural backgrounds under a single label can lead to misleading results. Consequently, considerable discussion has been conducted on the value of various approaches to ethnic identification during subject selection and sampling. For example, researchers have identified Hispanics based on family surname, birthplace, use of the Spanish language, and “Spanish origin.” Each of these methods has inherent advantages and disadvantages (e.g., Hayes-Bautista 1980), and the existing evidence indicates that the different methods generate noncomparable samples. Some researchers also have disputed the use of the term “Hispanic,” considering it both misleading and racist (Hayes-Bautista 1980).

Ethnic identification in alcohol research has been based primarily on self-identification. Typically, participants are presented with several categories identifying all ethnic groups in the United States and are asked to choose the category that best describes their ethnic identification. A category labeled “Other” is provided for people who do not feel comfortable placing themselves in any of the specific ethnic categories provided. People who select the “Other” category usually are asked to describe their ethnic identity.

It is also important to collect other types of information associated with ethnicity and culture of origin. For example, in the National Alcohol Surveys of 1984, 1992, and 1995, all respondents were asked for their mother’s birthplace, their father’s birthplace, the country of origin for most of their ancestors, their own birthplace, and the number of years they had lived in the United States.

Researchers also have attempted to assess intraethnic diversity in alcohol use, using various approaches, including the following:

- Hispanics have been classified according to their national origin, acculturation level, immigration/ generational status, and socioeconomic status.
- Blacks have been subgrouped based on their internal migration history, the region of the country where they were interviewed, and their socioeconomic status.
- Asian-Americans have been described based on their acculturation status and national origin.
- Native American studies have been conducted with regard to tribal affiliation and residence (i.e., whether they live on a reservation or in an urban area).

***Subject Selection***

Sampling strategies and the selection of subjects from minority groups require the consideration of intraethnic differences so that researchers can make informed, conscious decisions about sample composition. Thus, investigators must decide whether to conduct their research only in a particular subgroup (e.g., only Puerto Ricans) or in a sample representing all subgroups of the ethnic groups under consideration (e.g., all Hispanics). Focusing on one national subgroup allows researchers to control for the effect of country of origin, a variable often used as a proxy for national culture. For example, researchers recently analyzed the influences of acculturation and cultural attitudes on drinking and driving among Hispanics in California. In order to base their conclusions on people with a rather uniform set of values and attitudes but across a continuous spectrum of acculturation, the investigators decided to include only Mexican-Americans. One disadvantage of this approach, however, is that prolonged and expensive fieldwork may be necessary to sample adequate numbers of certain small but important subgroups (e.g., Cuban-Americans). This disadvantage is particularly relevant if the research is conducted in a part of the country where members of that particular subgroup are not well represented (e.g., Cuban-Americans in the Southwest of the United States).

When planning studies involving community samples (i.e., people in the general population), researchers can minimize the time and expense required by focusing on geographic areas with large concentrations of the population under study (e.g., Florida for studies of Cuban-Americans). For studies involving clinical samples (i.e., people receiving alcoholism treatment), researchers must identify and gain access to programs that cater to minority clients in order to recruit adequate sample sizes in the shortest time possible and at the lowest cost.

***Instrument Development***

Studies among ethnic minorities often include participants who are uncomfortable speaking English. Consequently, investigators must translate questionnaires and/or conduct their research in the native languages of the participants. The process of translating a questionnaire into another language has been described in detail elsewhere (Brislin 1986). At a minimum, this process involves the translation of the questionnaire into another language as well as translating the instrument back into the original language by an independent translator in order to compare the original and the “back-translated” forms for accuracy. Any discrepancies between the two forms must be discussed by the research team and solved by consensus.

In many situations, the process of questionnaire translation and back-translation involves a detailed and sometimes challenging search for terms or concepts that convey the same meaning in both languages. For instance, the concept of being “high” on alcohol or other drugs is difficult to translate into Spanish. For an international research project carried out in Mexico, Scotland, and Zambia, researchers used the Spanish translation of “feeling the effects of alcohol” to convey the concept of being “high” but not drunk. In another example, the condition identified as “alcohol withdrawal syndrome” by English-speaking researchers is called “abstinence syndrome” in Spanish.

Other difficulties arise when a behavior used to identify the presence of a disorder carries a negative connotation in one language but not in another language. For instance, tolerance to alcohol is considered an indicator of alcohol dependence in the English-language alcohol literature. In popular Mexican culture, however, tolerance to alcohol tends to have a positive meaning, indicating that a person is strong enough to drink large amounts of alcohol without showing the effects of drinking (Room et al. 1996). Such cultural differences have implications not only for the translation of data collection instruments but also for their cross-cultural validity.

Finally, it is crucial that cross-ethnic questionnaires cover issues of special interest to the ethnic groups under consideration. Such issues may include topics such as acculturation, years of life in the United States, family composition, ability to speak English as well as the native language, and experiences of discrimination and racism. To cover such topics, researchers may need to develop and test special questions to ensure their reliability and validity for people in the ethnic groups under study. The completion of those tasks requires expertise in developing instruments as well as additional expense and time for questionnaire preparation.

***Data Collection and Subject Recruitment***

Along with translating instruments, investigators must identify and train bilingual interviewers. Bilingual interviewers usually undergo a slightly longer training period than other interviewers because they must be familiar with both the English- and the foreign-language versions of the data collection instrument. This process again increases the costs associated with conducting research among minority groups. In addition, an interviewer’s ethnicity can influence the quality of data, especially when respondents are questioned about racially sensitive issues (Weeks and Moore 1981).

Finally, it is important to recognize that members of ethnic minorities often are unwilling to participate in research because of a general distrust of both government and authority. May (1982) has suggested that subject participation can be enhanced if members of ethnic minority groups perceive that the research has applied significance and if community leaders take an active role in the research process from the initial design to the dissemination of results.

***Conclusions***

Methodological aspects of research with ethnic minorities can make such studies more demanding and expensive to undertake than research on the majority population. Researchers should not avoid these challenges, however, and funding agencies should be prepared to recognize the methodological requirements and support this research. Already, all ethnic minorities combined constitute almost 25 percent of the U.S. population, and in some States (e.g., California), no ethnic group will have a clear majority by the year 2000. Consequently, public health researchers must understand drinking behavior and the development of alcohol problems among members of all ethnic groups. Policymakers also should take these issues into account and continue to pay special attention and provide support to the development of research with ethnic minority groups. Although special efforts (e.g., the recent set of principles adopted by the National Institutes of Health to guide research among women and minorities) have been undertaken recently by public health agencies to help develop research with ethnic minorities, this research still lags behind studies conducted with the majority population. The insights gleaned through the creation of complex models used to explain and predict drinking and alcohol-related problems among ethnic minorities will positively affect the health of all members of the U.S. population.

—Raul Caetano, Catherine L. Clark, and Tammy Tam

ReferencesBrislinRWThe wording and translation of research instrumentsLonnerWJBerryJWField Methods in Cross-Cultural Research8Newbury Park, CASage Publications1986137164Hayes-BautistaDEIdentifying “Hispanic” populations: The influence of research methodology upon public policyAmerican Journal of Public Health73353356198010.2105/ajph.70.4.353PMC16193946987907HeathDBUses and misuses of the concept of ethnicity in alcohol studies: An essay in deconstructionInternational Journal of the Addictions255A and 6A607628199110.3109/108260891090772632101395MayPASubstance abuse and American Indians: Prevalence and susceptibilityInternational Journal of the Addictions17118512091982675715510.3109/10826088209056349RoomRJancaABennettLASchmidtLSartoriusNWHO cross-cultural applicability research on diagnosis and assessment of substance use disorders: An overview of methods and selected resultsAddiction911992201996883527710.1046/j.1360-0443.1996.9121993.xWeeksMFMooreRPEthnicity-of-interviewer effects on ethnic respondentsPublic Opinion Quarterly452452491981

This article discusses the main theories advanced in the literature to explain drinking patterns and alcohol-related problems among the four major ethnic minorities in the United States (i.e., Hispanics, blacks, Asian-Americans, and Native Americans). For more detailed information on alcohol consumption and the associated consequences in each of those groups, the reader is referred to subsequent articles in this journal issue.

## Drinking Patterns and Underlying Causes Among Hispanics

The history of alcohol research among Hispanics in the United States exemplifies the difficulties in studying a heterogeneous minority population. Most analyses have treated Hispanics as a single group, despite the fact that traditional alcohol use patterns vary among Hispanics with different countries of origin. In addition, studies among Hispanics typically have focused on male drinking patterns. Although such studies are useful for providing an overview, they gloss over subgroup and gender differences in drinking patterns by referring to “standard” Hispanic cultural norms that promote male alcohol consumption and female abstention (for a review, see Aguirre-Molina and Caetano 1994).

More recent research, however, has demonstrated that drinking patterns and rates of alcohol-related problems often differ among Hispanic subgroups, as follows:

- According to the Hispanic Health and Nutrition Examination Survey (a large-scale survey of Hispanics residing in the Southwest; the Northeast; and Dade County, Florida), Mexican-American and Puerto Rican men have higher rates of heavy drinking than do Cuban-American men (see Aguirre-Molina and Caetano 1994).
- According to the same survey, Mexican-American women have higher rates of both abstinence and frequent heavy drinking than do Cuban-American and Puerto Rican women (see Aguirre-Molina and Caetano 1994).
- In other nationwide studies, Mexican-Americans exhibited more alcohol-related problems than did Cuban-Americans and Puerto Ricans (Caetano 1988).
- The prevalence of alcohol dependence is higher among U.S.-born Mexican-American women than among Puerto Rican and immigrant Mexican-American women (Canino et al. 1992).

One traditional explanation for heavy drinking patterns among Hispanic men, particularly Mexican-Americans, is the concept of “exaggerated machismo.” This concept, which has been neither well defined nor measured empirically, implies that Hispanic men strive to appear strong and masculine and that the ability to drink large amounts of alcohol exemplifies their masculinity. To date, however, no convincing association between “exaggerated machismo” and drinking patterns has been demonstrated. For example, in the 1984 National Alcohol Survey, the statement that “a real man can hold his liquor” was endorsed by 16 percent of Hispanic men, similar to the 13-percent endorsement rate by all American men. Another study using a more complex measure of machismo in a comparison of white, black, and Mexican-American men concluded that machismo was related to alcohol use among men regardless of ethnicity and could not explain the high drinking levels among Mexican-Americans (Neff et al. 1991).

Unfortunately, simple models relying on only one factor (e.g., machismo) to explain drinking patterns cannot account for the variations observed in drinking behaviors among Hispanics. To understand the complexity of alcohol use among members of that ethnic group, a multifactorial model is needed that takes into account social, economic, cultural, and historical aspects of Hispanic life in the United States. However, although information on sociodemographic and cultural determinants of drinking among Hispanics is available, little is known about historical variations in drinking among this ethnic group.

## Drinking Patterns and Underlying Causes Among Blacks

As with Hispanics, much of the discussion on alcohol consumption patterns among blacks (i.e., U.S.-born African-Americans as well as immigrants from the Caribbean, Africa, and Europe) has focused on the prevalence of heavy drinking and ignored patterns of abstention and lighter drinking. Drinking patterns among blacks traditionally have been thought to result from social disorganization (e.g., family breakdown and psychological dysfunction) (Herd 1987). Heavy drinking was considered a dominant characteristic of the “black” way of life, and early sociocultural studies characterized blacks’ attitudes toward alcohol as more permissive and liberal than those of whites. Furthermore, scholars have argued that alcohol advertising targeting the black community has promoted heavier alcohol consumption, particularly of malt liquor, among members of this ethnic group (Hacker et al. 1987; Herd 1993).

More recent examinations of historical trends and empirical data have provided a broader view of black drinking. For example, Herd (1987) demonstrated the importance of social change as a factor shaping blacks’ drinking patterns. In particular, the mass migration of blacks from the rural South to the northern areas of the United States that began in the early 1900s appears to have resulted in increased alcohol consumption. Results from the 1984 National Alcohol Survey showed that several sociodemographic factors (e.g., age) helped shape blacks’ drinking patterns, degree of alcohol-related problems, and attitudes toward drinking and that these influences differed from those observed among whites (Herd and Caetano 1987). For example, rates of heavy drinking among blacks were highest among men in their 40s and 50s, whereas the rates among whites were highest among men in their 20s. In the 1995 National Alcohol Survey, however, rates of heavy drinking among white men in their 20s have dropped, such that black and white men now show similar drinking patterns until they reach age 49. Moreover, in the 50-to 59-year-old age group, rates of heavy drinking are substantially higher among whites than among blacks (16 percent versus 3 percent, respectively). The causes of these changes in alcohol consumption patterns between 1984 and 1995 remain unclear. Finally, the abstention rate currently is higher among black men than among white men (36 percent versus 26 percent, respectively), further contradicting common assumptions about black’s drinking patterns (Caetano and Clark 1998*a*).

Other research also has indicated that the attitudes of blacks toward drinking and drunkenness are not overly permissive and, in some cases, tend to be more conservative than those of whites (Caetano and Clark 1998*c*). For example, many studies have documented relatively high abstention rates among black women compared with white women (Caetano and Kaskutas 1995; Herd and Caetano 1987). The results of the 1995 National Alcohol Survey further support those findings, with abstention rates of 55 percent among black women and 39 percent among white women (Caetano and Clark 1998*a*).

In summary, recent research has contradicted many of the stereotypes of alcohol consumption patterns among blacks. Most likely, blacks’ drinking patterns and alcohol-related problems result from a complex interplay of individual attributes, environmental characteristics, and historical and cultural factors that shape the life history of blacks in the United States. Alcohol researchers have begun to identify those factors and determine their relative importance.

## Drinking Patterns and Underlying Causes Among Asian-Americans

In contrast to Hispanics and blacks, Asian-Americans typically have been considered a “model minority,” with high rates of abstention and low rates of heavy alcohol use. This image likely results from the fact that few Asian-Americans enter alcoholism treatment and from the lack of research on alcohol consumption patterns among Asian-Americans who might be at risk for alcohol problems, such as refugees from Cambodia and Vietnam. Despite the generally low drinking rates among Asian-Americans, however, substantial variations in drinking behavior exist among different Asian subgroups. For example, in a study among four Asian ethnic groups in Los Angeles, there were more drinkers than abstainers among Japanese-Americans and Chinese-Americans and more abstainers than drinkers among Filipino-Americans and Korean-Americans (Chi et al. 1989; Kitano and Chi 1989). The rates of heavy drinking also differed greatly among Asian subgroups, with the highest proportions of heavy drinkers found among Japanese-Americans, followed by Filipino-Americans, Korean-Americans, and Chinese-Americans. Likewise, in an assessment of alcohol and other drug service needs among Asian-Americans in California, those of Vietnamese and Chinese-Vietnamese origin had higher alcohol consumption levels than did those of Japanese, Chinese, Korean, and Filipino origin (Sasao 1991). Overall, however, the lifetime alcohol use among all Asian-American subgroups in that study was lower than the national average.

Pronounced gender differences in alcohol consumption also exist among Asian-Americans, with Asian-American women being much more likely to abstain or consume lesser amounts of alcohol than their male counterparts (Chi et al. 1989; Kitano and Chi 1989). Again, substantial differences exist among drinking behaviors of various ethnic subgroups. Thus, drinking rates range from as high as 67 percent among Japanese-American women (Kitano and Chi 1989) and 52 percent among Cambodian-American women (D’Avanzo et al. 1994) to as low as 18 percent among Korean-American women and 20 percent among Filipino-American women (Kitano and Chi 1989). Researchers have developed several theories to explain the stereotyped drinking patterns of low alcohol consumption among Asian-Americans. A popular explanation is the flushing response that many Asians experience (Ewing et al. 1974; Johnson 1989; Zeiner et al. 1979). This response is a physiological reaction to alcohol ingestion that includes flushing of the skin, especially in the face and torso, and an increase in skin temperature. Other unpleasant symptoms associated with the flushing response include nausea, dizziness, headache, fast heartbeat, and anxiety. Various researchers have considered this physiological sensitivity to alcohol a protective factor against excessive alcohol use (Ewing et al. 1974; Johnson 1989). Some studies suggest, however, that flushing may only be protective for people who develop the flushing response rapidly after ingesting alcohol (Nakawatase et al. 1993; Park et al. 1984). Johnson (1989) further stated that flushing is associated with reduced drinking only for fast flushers (i.e., people in whom the flushing response occurs rapidly after alcohol ingestion) who live in intact Asian cultures with relatively liberal attitudes toward drinking, such as the Korean culture. Still other studies have detected no significant association between consumption level and the flushing response (Johnson et al. 1984). Finally, some researchers have argued that the unpleasant symptoms are related to the amount of alcohol consumed, not to flushing itself (Wilson et al. 1978).

Researchers also have argued that low alcohol consumption levels among Asians are related to cultural values, such as the influence of ancient Confucian and Taoist philosophies on Chinese and Japanese drinking styles. The emphasis on conformity and harmony in those philosophies is believed to promote a moderate drinking style (Singer 1974; Sue et al. 1985). Hsu (1981) suggested that the emphasis on responsibility to others in the Chinese culture helps reinforce moderate drinking and sanctions against drunkenness. Similarly, traditional Japanese culture focuses on interdependence, restraint, and group achievement and may thereby contribute to controlled drinking. Finally, drinking in most Asian cultures takes place in prescribed social situations, which may limit the likelihood of alcohol abuse (Kitano et al. 1985).

In accordance with such cultural influences on drinking behavior, acculturation to mainstream American culture would be expected to cause Asians to adopt white drinking patterns. In fact, some studies have shown that later generations of immigrants tend to perceive more relaxed Asian cultural norms and to drink more than do their parents (Li and Rosenblood 1994). Furthermore, Asians born in the United States have higher rates of alcohol use than do Asians born in their ancestral homelands (Johnson et al. 1987). Other studies, however, did not confirm these cultural explanations. For example, Akutsu and colleagues (1989) found that the level of acculturation did not significantly predict alcohol consumption among Asian-Americans when the effects of physiological reactivity (i.e., flushing) were controlled. Likewise, Chin and colleagues (1991) found that most male alcoholics in New York’s Chinatown were Chinese immigrants who had had very limited exposure to the mainstream culture. One should note, however, that in the past several decades many Asian countries (e.g., Korea, Taiwan, and Hong Kong) have undergone fast economic growth, accompanied by rapid urbanization and the adoption of a more Western lifestyle. Consequently, Western values may have influenced the traditional value structures found in those Asian societies and diminished the impact of traditional social controls on alcohol consumption.

More recently, researchers have begun to evaluate stress and social adjustment as factors contributing to drinking behavior among Asian immigrants. Studies have shown that recent immigrants experience stress arising from economic hardship, occupational problems, acculturation difficulties, and social isolation (Lin et al. 1984). Exposure to such stressors may contribute to increased alcohol use. In a study of Southeast Asian refugees, Yee and Thu (1987) found that approximately 45 percent of respondents reported having problems with alcohol use, and a large proportion of the sample considered alcohol use an acceptable way to cope with stressful situations. Similarly, 45 percent of a sample of Cambodian refugee women acknowledged using alcohol for self-medication for stress and pain (D’Avanzo et al. 1994). Most likely, no conceptual model that focuses on only one facet of the phenomenon can account for the complex drinking patterns among Asian-Americans. Future research should take an integrative approach to address the differences among various Asian-American ethnic groups and to identify the interactive effects of the physiological, cultural, and social factors specific to each subgroup.

## Drinking Patterns and Underlying Causes Among Native Americans

As with Hispanics and blacks, much of the literature on Native American alcohol consumption has focused on heavy drinking or binge drinking. Many of the discussions are based on the “Fire-water Myth,” which suggests that Native Americans are predisposed to heavy alcohol consumption and are unable to control their drinking and their behavior when intoxicated (Mail and Johnson 1993). This myth dates back to the late 1600s, when British settlers, French trappers, and other colonial observers in North America (including Benjamin Franklin, the Jesuits, and members of the Lewis and Clark exploration party) noted the presumed insistence of Native Americans on drinking to the point of intoxication and the resulting alcohol-induced debauchery and violence (Duran and Duran 1995; Mancall 1995). That myth still persists, and many people, including many Native Americans, still consider heavy binge drinking to be representative of the “Indian way of drinking” (Duran and Duran 1995).

However, as with single-variable explanations of alcohol consumption patterns among other ethnic groups, the Firewater Myth is insufficient to describe and explain drinking among Native Americans for two main reasons. First, no evidence exists to demonstrate increased physiological or psychological reactivity to alcohol among Native Americans compared with other ethnic groups (Garcia-Andrade et al. 1997). Second, Native Americans are a highly heterogeneous ethnic group of more than 500 tribes who speak more than 200 distinct languages. Alcohol use varies widely among those tribes (Mancall 1995). For example, the Navajo tend to view social drinking as acceptable, whereas the Hopi consider drinking irresponsible (Mail and Johnson 1993). In fact, many Native Americans abstain from alcohol use, and Duran and Duran (1995) argue that “[t]emperance has either been the major focal point or a dominant theme in many native social movements over the last two hundred years” (p. 126). Some Native Americans are lifetime abstainers, and others quit drinking during early and middle adulthood as they take on family and tribal responsibilities (Mail and Johnson 1993).

Still, some Native Americans do engage in heavy and dangerous alcohol consumption, and numerous hypotheses have attempted to explain this phenomenon. Some theories focus on societal factors, such as poverty, unemployment, lack of opportunity, and lack of integration into either traditional Native American or Western culture. Other theories posit that Native Americans drink to cope with various negative emotions, including low self-esteem, anxiety, frustration, boredom, powerlessness, isolation, hopelessness, and despair. Finally, some theories are more specific to Native American culture, suggesting that Native Americans drink rapidly to induce an altered state of consciousness, a practice congruent with some traditional Native American practices (Duran and Duran 1995; Garcia-Andrade et al. 1997; Mail and Johnson 1993; Mancall 1995).

Unfortunately, no systematic nationwide surveys have assessed alcohol use among Native Americans. Accordingly, it is difficult to adequately describe current patterns of alcohol consumption among the various tribes or to test theories that have been advanced to explain Native American drinking patterns.

## Conclusions

The study of alcohol consumption among ethnic minorities in the United States has been maturing in recent years. Researchers in the field are moving away from single-factor explanations of drinking and are beginning to develop and test theories focusing on the complex interplay of psychological, historical, cultural, and social factors that describe and explain alcohol use among minority groups. Likewise, other factors—for example, sociodemographic characteristics (e.g., age, income, education); attitudes toward drinking; norms regulating drinking behavior; and alcohol availability as determined by taxation, number of alcohol outlets in the community, and hours of sale—likely predict various kinds of ethnic-specific drinking patterns. By recognizing the heterogeneity within each ethnic group, it will be easier for researchers and clinicians to identify the subpopulations that are truly at risk and which should be targeted by prevention and intervention programs (Mail and Johnson 1993). Furthermore, researchers must publicize factual reports of the drinking behaviors of ethnic minority groups, so that inaccurate stereotypes—such as the “macho Hispanic” and “drunken Indian”—are not perpetuated. Such stereotypes continue to undermine efforts of ethnic communities to find acceptance in society at large.

## Footnotes

1 Acculturation is defined as the adaptation to or acquisition of the beliefs and values of the dominant culture.
